# Pseudohypoparathyroidism type 1 B mimicking Fahr’s disease in a 28‐year‐old female: A case report

**DOI:** 10.1002/ccr3.5418

**Published:** 2022-02-06

**Authors:** Suman Acharya, Sushil Kumar Yadav, Gaurav Nepal, Siddhartha Bhandari, Shiva Lal Bhattarai, Santosh Pathak, Bipin Kandel, Jeevan Gautam, Roshan Bhandari

**Affiliations:** ^1^ 113015 Department of Internal Medicine, Maharajgunj Medical Campus Tribhuvan University Institute of Medicine Kathmandu Nepal

**Keywords:** parathyroid hormone resistance, PHP 1B, pseudohypoparathyroidism type 1B, PTH resistance

## Abstract

In virtue of precise clinical history, physical examinations, and biochemical/radiological investigations, pseudohypoparathyroidism can be effectively diagnosed, and its types can be differentiated even without exorbitant tests.

## Graphical abstract

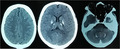

## 1 INTRODUCTION

Pseudohypoparathyroidism (PHP) is a disorder of hormonal signaling through G_s_α (stimulatory G protein) linked PTH receptor, and it causes PTH resistance by altering either G_s_α function or the function downstream of the signaling pathway. PHP can be either due to autosomal dominant mutation or due to epigenetic alteration in the expression of certain genes namely, GNAS, PRKAR1A, and PDE4D. The major clinical and laboratory features of PHP are AHO (Albright's hereditary osteodystrophy), hypocalcemia, hyperphosphatemia, and increased intact parathyroid hormone (iPTH).

The prevalence of PHP in Japan and Denmark was estimated to be 0.34/100,000 and 1.1/100,000, respectively, while the exact worldwide prevalence is not known.^1^ We present a case of pseudohypoparathyroidism type 1B (PHP 1B) with a 4‐year history of spasms of all the limbs. We could identify only four cases of PHP 1B,^2^, ^3^, ^4^, ^5^ which resembled this case clinically, in the Medline literature search. Moreover, a computed tomography (CT) scan revealing calcification of bilateral basal ganglia, frontal subcortical white matter, thalami, and dentate nuclei make this case resemble Fahr's disease and such resemblance is rare.^6^

## 2 CASE DESCRIPTION

A 28‐year‐old, average‐built female patient, who was having spasms of all four limbs for the last four years, presented with an increase in severity of spasms for the last two weeks. Her spasms involved abdominal muscles and limbs. When she visited our center, she was having 3–4 episodes of spasms per day with each episode lasting about 20 min on average. There was no history of loss of consciousness, headache, visual problems, fever, nausea, vomiting, shortness of breath, chest pain, altered sensorium, trauma, focal weakness, dyspnea, or abdominal pain. On examination, the patient was well oriented to time, place, and person. She had a blood pressure of 120/100 mmHg, respiratory rate of 24 breaths per minute, oxygen saturation at room air of 97%, heart rate of 90 beats per minute, and random blood glucose of 144 mg/dl. There was no pallor, icterus, lymphadenopathy, cyanosis, or clubbing. Trousseau sign was present; however, other neurological, cardiovascular, pulmonary, and abdominal examinations were unremarkable. Laboratory findings of the patients done after inpatient admission are shown in Table 1.

**TABLE 1:** Various relevant laboratory findings of our case

Parameters	Results	Reference (Units)
Serum		
Total calcium	1.3 mmol/L	2.1–2.6 mmol/L
Inorganic phosphorus	5.4 mg/dl	2.5–4.8 mg/dL
Magnesium	1.2 mg/dl	1.7–2.5 mg/dL
Potassium	3.4 mEq/L	3.5–5.2 mEq/L
Sodium	135 mEq/L	135–146 mEq/L
Albumin	46 U/L	37–47 U/L
Intact parathyroid hormone	291 pg/ml	7.5–53.5 pg/ml
Vitamin D	39.3 ng/ml	30–100 ng/ml
Urine		
24 h urine calcium	0.5 mmol/day	2.5–7.5 mmol/day
24 h urine phosphorus	275.7 mg/day	400–1300 mg/day

**FIGURE 1:**
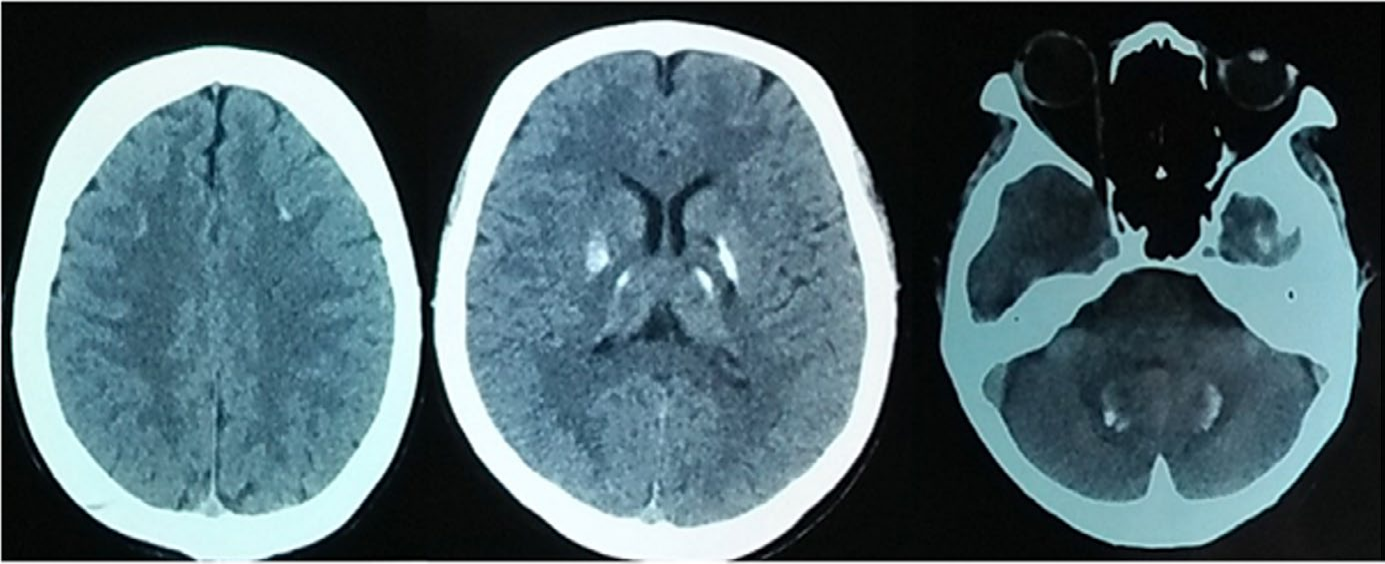
Computed tomography scan of the head showing multiple coarse calcific foci in bilateral frontal subcortical white matter, basal ganglia, thalami, and dentate nuclei

Ultrasonogram of abdomen and pelvis and electrocardiogram were normal. CT scan of the head revealed multiple coarse calcific foci in bilateral frontal subcortical white matter, basal ganglia, thalami, and dentate nuclei (Figure 1).

Based on history, examination, and the presence of hypocalcemia, hyperphosphatemia, elevated serum iPTH level, and a normal vitamin D level, a diagnosis of PHP was made. Due to the limited resources and financial constraints, the genetic basis for PHP could not be established and a PTH infusion test could not be performed. The absence of Albright's hereditary osteodystrophy (AHO) and the presence of features of PTH resistance with cerebral calcification but without neurocognitive impairment indicate PHP1B in this patient.

The patient was treated for hypocalcemia by vitamin D 60,000 IU capsules and calcium gluconate infusion. After 3 days, blood calcium level was back to normal and symptoms disappeared, and calcium gluconate infusion was replaced by oral calcium‐vitamin D supplements. At 3 months follow‐up, the patient is doing well, spasms have not recurred, and the patient has a good quality of life.

## 3 DISCUSSION

Parathyroid hormone and vitamin D along with calcitonin control calcium distribution within the human body. Parathyroid hormone and vitamin D increase blood calcium level by increasing its absorption from the gut and kidney and by increasing bone resorption.^7^ PTH resistance is clinically defined by hypocalcemia, hyperphosphatemia, and increased PTH level in the presence of normal vitamin D level, blood magnesium level, and urinary function.^1^ The biochemical findings of our case are consistent with the definition of PTH resistance. The absence of common causes of secondary hyperparathyroidism that is, chronic kidney disease, Vitamin D deficiency, gastric or intestinal surgery, and chronic diarrhea^8^ and the presence of features of PTH resistance and brain calcifications supported the diagnosis to be pseudohypoparathyroidism.^6^, ^9^ PTH resistance is seen in PHP and PHP‐related disorders. PHP and its related disorders are classified into PHP1A, PHP1B, PHP1C, PHP2, PPHP (pseudopseudohypoparathyroidism), POH (progressive osseous heteroplasia), and acrodysostosis (types 1 and 2) based on clinical and laboratory features. The main manifestation of the above‐mentioned conditions, except PHP1B, is AHO (Albright's hereditary osteodystrophy), which is characterized by a round face, stocky habitus with short stature, brachydactyly, and ectopic ossification. Cerebral calcification is seen in both PHP1A and PHP1B, but PHP1A also presents with multiple hormonal resistances and cognitive impairment while PHP1B is an isolated resistance to PTH without any cognitive impairment reported.^1^ The absence of AHO and neurocognitive impairment and the presence of features of PTH resistance and cerebral calcification indicate PHP1B in this patient.

PHP often presents with radiological (bilateral calcification involving basal ganglia, dentate nucleus, and other parts of the brain) and clinical features (movement disorders and cognitive changes) resembling Fahr's disease, but psychiatric features are absent in PHP, which is common in Fahr's disease. The absence of psychiatric features in PHP also differentiates it from other parathyroid disorders because psychiatric symptoms are common in hypoparathyroidism and other parathyroid disorders.^6^ A total of 55% of patients with Fahr's disease present with movement disorders, the most common being Parkinsonism. Cognitive impairment, cerebellar, and speech disorders are other manifestations of Fahr's disease.^9^ Seizure is uncommon in the case of Fahr's disease, whereas it is present in most cases of PHP. Thus, the presence of seizures helps differentiate PHP from Fahr's disease.^6^ Although seizure was not present in this case, the presence of features of PTH resistance and absence of movement disorders and cognitive impairment differentiate it from Fahr's disease.

Calcification in Fahr's disease is because of either SLC20A2 or PDGFRB gene mutation. The former encodes sodium‐dependent inorganic phosphate transporter 2, which is essential for cellular calcium and phosphate homeostasis, and the latter encodes receptors for platelet‐derived growth factor, which are essential for maintaining blood–brain barrier.^6^ Calcification in case of pseudohypoparathyroidism is due to loss of function mutation on the maternal allele of GNAS.^6^

We identified and reviewed four case reports of PHP1B, which presented similarly to this case. The detailed comparison of previously reported case with ours is tabulated in Table 2.

**TABLE 2:** Comparison of published cases of pseudohypoparathyroidism type 1B with our case

Case reference	Age (years)	Sex	Calcification in brain	Clinical signs and symptoms
Present case	28	Female	Frontal subcortical white matter, basal ganglia, thalami, and dentate nuclei	Tetany, abdominal muscle spasms, Trousseau's sign present
Garcia et al. 2018^3^	16	Male	Basal ganglia, subcortical frontal lobe, and subcortical parietal lobe	Tetany, involuntary dystonic movement of left hand
Garg et al. 2011^4^	34	Male	No calcification	Recurrent carpopedal spasms, perioral numbness, cramps, muscle twitching, generalized weakness, trousseau's sign present, Chvostek's sign present
Kutilek et al. 2018^5^	16	Male	Basal ganglia	Tetany, Chvostek's sign present
Yang et al. 2020^2^	38	Male	Basal ganglia, frontal lobe, cerebellar hemisphere	Seizure, tetany, trousseau's sign present, Chvostek's sign present

All the cases presented in the Table above responded well to therapy that is, calcium and vitamin D supplementation. Sodium valproate was used in the case of Yang et al. to treat seizures before the diagnosis of PHP, but the anticonvulsant therapy was not found to be effective.^2^ There are other cases of PHP as well in which seizures did not respond well to anticonvulsants administered without calcium and vitamin D supplementation.^10^ A case of PHP reported by Kwon et al.^11^ presented with paroxysmal kinesigenic dyskinesia did not have brain calcification as in a case reported by Garg et al.^4^ Unresponsiveness of seizure to anticonvulsants and the presence of abnormal involuntary movements even without brain calcification suggest hypocalcemia as the major factor behind these manifestations. This is also supported by the fact that these symptoms are well controlled after normalizing serum calcium levels.

## 4 CONCLUSION

In virtue of precise clinical history, physical examinations, and biochemical/radiological investigations, PHP can be diagnosed, and its types can be differentiated even without exorbitant genetic testing or PTH infusion test. In resource‐limited setting, PHP can easily be misdiagnosed as Fahr's disease which has an entirely different line of management. Hypocalcemia can be the major cause behind spasms, convulsions, and movement disorders in PHP rather than brain calcification, and these features can be effectively controlled by maintaining a normal blood calcium level.

## CONFLICT OF INTEREST

The authors declare that they have no competing interests.

## AUTHOR CONTRIBUTIONS

RB, SA, and GN were involved in patient care (diagnosis, treatment, and follow‐up). SKY, SB, BK, SLB, JG, and SP contributed to the collection of case information, writing of the manuscript, and manuscript revision. GN, RB, SA, and SB were involved in revising the manuscript critically for important intellectual content. All authors approved the final version.

## ETHICAL APPROVAL

This study did not include experiments on animals or humans.

## CONSENT

Written informed consent was obtained from the patient for the publication of this case report and any accompanying images. A copy of the written consent is available for review by the Editor‐in‐Chief of this journal.

## Data Availability

The data used in the case report are available on reasonable request.
